# Pre-diabetes is associated with attenuation rather than volume of epicardial adipose tissue on computed tomography

**DOI:** 10.1038/s41598-023-28679-w

**Published:** 2023-01-28

**Authors:** David Molnar, Elias Björnson, Måns Larsson, Martin Adiels, Anders Gummesson, Fredrik Bäckhed, Ola Hjelmgren, Göran Bergström

**Affiliations:** 1 Department of Molecular and Clinical Medicine, Institute of Medicine, Sahlgrenska Academy, University of Gothenburg, Box 428, 40530 Gothenburg, Sweden; 2 Department of Radiology, Sahlgrenska University Hospital, Region Västra Götaland, Gothenburg, Sweden; 3 Eigenvision AB, Bredgatan 4, 211 30 Malmö, Sweden; 4 Sahlgrenska Academy, and School of Public Health and Community Medicine, Institute of Medicine, University of Gothenburg, Gothenburg, Sweden; 5 Department of Clinical Genetics and Genomics, Sahlgrenska University Hospital, Region Västra Götaland, Gothenburg, Sweden; 6 Novo Nordisk Foundation Center for Basic Metabolic Research, Section for Metabolic Receptology and Enteroendocrinology, Faculty of Health Science University of Copenhagen, Copenhagen, Denmark; 7 Department of Clinical Physiology, Sahlgrenska University Hospital, Region Västra Götaland, Gothenburg, Sweden

**Keywords:** Type 2 diabetes, Predictive markers, Body mass index, Computed tomography, Risk factors, Software, Atherosclerosis, Diabetes, Obesity, Pre-diabetes

## Abstract

The volume of epicardial adipose tissue (EATV) is increased in type-2 diabetes (T2D), while its attenuation (EATA) appears to be decreased. Similar patterns have been suggested in pre-diabetes, but data is scarce. In both pre-diabetes and T2D, any independent role of EATV and EATA in disease development remains to be proven, a task complicated by their substantial co-variation with other anthropometrics, e.g. BMI, waist circumference, and abdominal visceral adipose tissue (VAT). EATV and EATA was quantified in computed tomography (CT) images in a population study (n = 1948) using an automatic technique. Data was available on BMI, waist circumference, abdominal visceral adipose tissue (VAT) area, insulin resistance (IR) and glucose tolerance, the latter ranging from normal (NGT), over pre-diabetes (impaired fasting glucose [IFG, n = 414] impaired glucose tolerance [IGT, n = 321] and their combination [CGI, n = 128]), to T2D. EATV was increased in pre-diabetes, T2D and IR in univariable analyses and when adjusting for BMI, however not when adjusting for waist or VAT. EATA was reduced in pre-diabetes, T2D and IR in univariable analyses and when adjusting for BMI and waist, however not when adjusting for VAT. Adjustment for other co-variates had little influence on the results. In conclusion, EATV is increased and EATA reduced in pre-diabetes, T2D and IR, however, significant co-variation with other anthropometrics, especially VAT, obscures their function in disease development. The current results do not exclude a pathophysiological role of epicardial fat, but future studies need to adjust for anthropometrics, or focus on the microenvironment within the pericardial sac.

## Introduction

Type-2 diabetes (T2D) has become one of the leading causes of morbidity globally, with more than 450 million cases diagnosed, projected to increase further over the next decades^1^. Various stages of pre-diabetes are believed to reflect the gradual changes in glucose and insulin metabolism^2,3^ leading to T2D. These range from minimal hypersecretion of insulin in overweight normoglycaemic individuals over reduced glucose tolerance and impaired fasting glucose to finally T2D. Pre-diabetes and T2D are strongly associated with obesity, but all fat depots are not created equal, exhibiting various profiles of insulin sensitivity, lipolytic and secretory activity^4^. The visceral fat depots have been suggested as plausible culprits^2,3^. One of these, the epicardial adipose tissue (EAT), has attracted much interest lately, as it might be of relevance in the pathogenesis of coronary atherosclerosis in glucose disorders^5–7^. Increased inflammatory activity has been found in EAT in patients with coronary artery disease^8,9^, and there is also data supporting increased inflammatory activity of EAT in T2D^10^. Little is known about EAT in pre-diabetes in this context.

EAT volume (EATV) seems to be increased in pre-diabetic individuals^5,11,12^, but to a lesser extent than in individuals with T2D, consistent with a gradual disease progression. The relationship between the various stages of pre-diabetes and EATV has not previously been well described, and the published cohorts are small. Increased EAT in T2D is better documented, both with echocardiography^13–15^ (thickness) and computed tomography (CT)^5,16–18^ (actual volume). Increased EATV has been both suggested^19–21^ and refuted^5,22^ as an independent risk factor for coronary artery disease in T2D, but the link between the conditions has not been fully elucidated. The strong co-variation between EATV and other anthropometric factors, such as BMI, waist circumference and visceral adipose tissue (VAT)^23,24^ is a complicating factor in discerning an independent role of EATV in coronary pathophysiology.

The attenuation of EAT (EATA), or its measured radiodensity in Hounsfield units (HU) on CT, is a function of its composition, with lower values generally indicating a higher relative lipid content. In a study by Franssens et al., EATA was decreased in T2D and decreased with increasing insulin resistance^25^, i.e. increasing derangement of glucose metabolism. The finding of decreased EATA among T2D patients was confirmed in a much larger recent study by Milanese^17^. This could point to epicardial lipid accumulation in T2D, however, there are some possible confounders. It is known that an increase in metabolically active brown adipose tissue can increase EATA^26,27^, something which can be seasonally dependent^28^. Increases in EATA could also be an effect of increased cellularity due to inflammation or an increase in extracellular matrix seen in fibrotic (post)inflammatory conditions^29^. EATA has reported to be lower in women, and lower with advancing age^25,30^, the latter possibly due to a reduction of brown adipose tissue^31^. Finally, cholesterol metabolism could influence EATA independently of subcutaneous adipose tissue^32^. Unfortunately, not much data is available on EATA in large cohorts of pre-diabetic individuals, and no study has yet reported data adjusted for relevant anthropometric measures.

Much of the research on EAT has until recently been hampered by the lack of automated methods for reliably quantifying EATV and EATA. Apart from the application of an automatic model developed by Commandeur et al.^33^ in a study focusing on cardiac events (n = 2068)^34^ little has been published on automatic measurement of EAT parameters in larger cohorts. We have developed a fully automatic model with excellent metrics^24^ for non-contrast enhanced cardiac CT images. The model is capable of segmenting the entire EATV in both noisy and/or incomplete images to specifically target large cohort studies at population level. In this work, cross-sectional data from the IGT study (n = 1948) was used to test whether EATV and EATA is altered in pre-diabetes, T2D and insulin resistance, and if so, independently of changes in anthropometric measures (BMI, waist and visceral adipose tissue [VAT]).

## Materials and methods

### Population

Data from participants in the “Microbiota, development of type 2 diabetes and cardiovascular disease study” (IGT-study) was used. Participants in the IGT-study were recruited among men and women born in Sweden and aged 50–64 years, who were randomly selected from the Swedish population registry. Initial contact was made by sending out an information brochure asking the recipient to contact the research group for a screening visit.

A fasting capillary glucose test and an oral glucose tolerance test (OGTT) with capillary glucose taken 120 min after 75 g of oral glucose load was performed in accordance with WHO methodology and criteria^35^. Individuals fulfilling the criteria for either T2D (type 2 diabetes), IFG (impaired fasting glucose), IGT (impaired glucose tolerance) or CGI (combined glucose intolerance) were eligible for inclusion. Individuals were also eligible for inclusion regardless of the glucose test results, if they showed an increased risk for future diabetes according to the Finnish Diabetes Risk Score (FINDRISC, score > 14)^36^, or had 2 first degree relatives with T2D. Individuals with normal glucose tolerance (NGT) and no increased risk of developing T2D according to the FINDRISC questionnaire (≤ 14) were randomized (1:4) to inclusion as controls.

Previously known diabetes, i.e. prior to doing the screening above, was an exclusion criterion in order to ensure that no effects from any previously received treatment would interfere with results. Other severe diseases, e.g. inflammatory bowel disease, rheumatic diseases, malignancy (unless no relapse during 5 years of follow-up), treatment with steroids or immune-modulating treatment, pharmacological treatment of infection during the last 3 months and major cognitive dysfunction were also exclusion criteria.

A total of 1965 subjects were included in the IGT-study, and from these a total of 1948 individuals had retrievable CT images, and were included in the analyses of the current study, comprising the subgroups NGT, IFG, IGT, CGI and T2D.

The ethics committee at Gothenburg University approved the study (Dnr 560-13) which was conducted in accordance with the Declaration of Helsinki. Participants gave written informed consent.

### Anthropometry and blood pressure

Weight was measured to the nearest 0.1 kg on digital SECA 910 electronic scales (Vogel and Halke, Hamburg, Germany), with the subjects in light clothing. Body height and waist circumference were measured according to current recommendations^37^.

### Questionnaires

A questionnaire was used to collect detailed information on self‐reported health, smoking, alcohol habits, current medication, leisure-time moderately vigorous physical activity (MVPA), and education level.

### Insulin resistance

Insulin was measured in venous fasting blood samples using ELISA (Mercordia AB Uppsala Sweden). Insulin resistance was estimated using the HOmeostatic Model Assessment for Insulin Resistance (HOMA-IR) using the following formula: (HOMA-IR) = ([insulin × capillary glucose] / 22.5).

### Imaging

All imaging was performed using the same CT-scanners and protocols, Siemens Somatom Definition Flash with a Stellar detector (Siemens Healthcare, Forchheim, Germany). Care Dose 4D was used for dose optimization. Cardiac image acquisition was ECG-gated, with tube voltage of 120 kV, and refmAs of 80. These images have a matrix of 512 × 512 voxels in the axial plane, with a square DFOV in the range of 170–200 mm and were reconstructed using the B35f. HeartView medium CaScore algorithm, generating a slice thickness of 3 mm, with 50% overlap between slices. Images to analyze subcutaneous and abdominal VAT depots were acquired as one image with a slice thickness of 5 mm at the level of the fourth lumbar vertebra (L4).

### Analysis of EATV and EAT attenuation

Non-contrast cardiac CT examinations from totally 1948 individuals were analyzed automatically for EATV and median EAT attenuation with a deep-learning based model previously developed by our group^24^. In short, the model, consisting of two neural networks in tandem, works by: identifying the pericardium and the heart chambers (Dice-score 0.90), measuring the volume and attenuation values of all voxels inside the pericardium but outside the heart chambers corresponding to adipose tissue (-30 to -190 HU), and finally, in any cases of incomplete image stacks, predicting the correct values of EATV with high precision, based on image features.

### Automatic quality check of segmentations

Based on experiences during the development of the method, it was clear that failed segmentations would be expected in a limited number (~ 0.6%). In order to reduce the need for manual proofreading, we developed a feature whereby potentially failed segmentations are automatically flagged by the software in order to be reviewed, and if needed, manually corrected.

The method to identify failed segmentations was developed through detailed reviewing of 1400 automatically segmented CT examinations by an expert thoracic radiologist (author DM) with regards to segmentation quality. A total of 8 failed segmentations were identified (~ 0.6%), which all showed very conspicuous errors in their 3-D shape, erroneously including or omitting significant volumes of adipose tissue. We postulated that any failed segmentation would deviate significantly from the normal anatomic shape of a heart, and the following operations are now included in the analytical work-flow as at the final stage: (i) extraction of the volume ”heart”, (ii) shrinkage of it with 15 mm in all dimensions, (iii) removal of everything except its largest component, (iv) growing of the largest component by 35 mm in all dimensions to create a mask (v), which if smaller than the original segmentation, will flag the segmentation as potentially failed. The volume of the segmentation outside of the mask is calculated and tabulated in the output of the final model. In order to validate the performance of the automatic identification of failed segmentations and to find a suitable cut-off for volumes outside the mask, 400 CT examinations from the cohort used in the current study were manually reviewed in detail. A total of 3 cases of failed segmentations were found, which were all correctly flagged as potentially failed. A cut-off was selected based upon this, and previous rounds of manual quality-checking, such that no truly failed segmentations would be missed, and falsely flagged segmentations would be kept at a reasonable level for manual work-up.

### Analysis of abdominal adipose tissue

The area of abdominal fat was segmented as previously described by Kullberg et al.^38^ using thresholding followed by applying a purpose built inside lean-tissue filter to separate visceral adipose tissue (VAT) from subcutaneous adipose tissue (SAT) by identifying regions in the image that are inside lean tissue. Median attenuation values of VAT and SAT were calculated.

### Statistics

Data from the study participants was ordered in five groups: NGT, IFG, IGT, CGI and T2D. Data was analyzed as complete cases, without imputation. Descriptive data on the cohort is presented as median values with interquartile ranges. The Pearson Chi-square test was performed to assess differences in categorical variables between the groups, while the Kruskal–Wallis independent samples test was performed to assess differences in continuous variables between the groups, p < 0.05 being considered significant in both tests. Kendall’s Tau coefficient was used to test the correlation between changes in EATV and EATA and the glucose groups in the order NGT-IFG/IGT-CGI-T2D, p < 0.05 being considered significant. To assess the degree of co-linearity between different anthropometrics, the correlations between BMI, waist circumference, VAT area and EATV or EATA was estimated in the NGT group. The NGT group was also used to select relevant confounders to adjust for in the regression analyses evaluating the following potential confounders: sex, age, level of physical activity, treatment for hyperlipidemia, smoking, alcohol use, level of education and season for CT examination. Confounders which showed an association with EATV independently of VAT area (p < 0.10) were qualified. We then used five different linear regression models to identify whether any of the different pre-diabetes groups, the T2D group or HOMA-IR were independently associated with EATV. In model 1, the pre-diabetes groups, the T2D group and HOMA-IR were tested on their own for association with EATV. In model 2–4, other anthropometric data were added to the model one by one (BMI, waist and VAT area) to identify whether the associations observed in Model 1 were independent of concomitant changes in other anthropometrics. In Model 5, the remaining confounders were introduced. Five similar regression models were used to analyze EATA with the addition of a model testing whether the association was independent of EATV. A p-value < 0.05 was considered significant.

## Results

### Data integrity

Among the 1948 participants with available cardiac CT imaging data included in the study, there were four cases with missing data on VAT (Table 1). Thus, totally 1944 participants were included in the statistical analyses of EAT and VAT data versus different glucose groups. Among these there were 28 cases of missing HOMA-IR data, distributed among all glucose groups, reducing the number of participants included in the analyses of EAT and VAT data versus HOMA-IR to 1916.

**Table 1:** Characteristics of the studied cohort (total n = 1948) with the five subgroups shown in the columns.

	NGT (n = 1012)	IGT (n = 321)	IFG (n = 414)	CGI (n = 128)	T2D (n = 73)
Sex, male*	61.7%	56.4%	43.0%	43.0%	37.0%
Age, years	57.5 (7.8)	60.5 (8.6)**	58.4 (7.8)	60.4 (6.2)**	60.0 (6.9)**
Smokers	8.3%	7.8%	9.2%	10.2%	12.3%
Education level*					
Elementary	7.0%*	13.1%*	6.1%*	10.9%*	13.7%*
High-school	45.8%*	52.3%*	52.2%*	46.9%*	52.1%*
University	47.2%*	34.6%*	41.7%*	42.2%*	34.2%*
Physical activity (moderately vigorous)	4.7%	4.3%	4.9%	3.9%	3.9%
Alcohol use (AUDIT)					
Low	4.2%	7.5%	2.9%	3.9%	6.8%
Low-moderate	13.6%	12.1%	11.8%	20.5%	19.2%
Moderate	42.0%	35.5%	39.4%	40.2%	46.6%
Moderate-high	35.1%	35.8%	39.4%	27.6%	17.8%
High	5.1%	9.0%	6.5%	7.9%	9.6%
Season for CT					
Winter	26.2%	23.1%	26.3%	25.0%	26.0%
Spring	30.3%	29.9%	29.2%	32.8%	43.8%
Summer	12.4%	15.3%	10.9%	9.4%	8.2%
Fall	31.0%	31.8%	33.6%	32.8%	21.9%
Lipid lowering medication*	7.6%	12.1%	6.8%	9.4%	26.0%
HOMA IR	1.33 (1.0)^a^	1.48 (1.4)^a^	1.73 (1.3)^a^**	2.70 (2.5)^a^**	3.22 (2.2)^a^**
BMI, [kg/m^2^]	26.8 (5.7)	26.9 (6.1)	27.1 (5.1)	29.9 (6.5)**	30.6 (7.8)**
Waist, [cm]	96 (16)	97 (20)	98 (15)**	105 (16)**	107 (19)**
Abdominal VAT area, [cm^2^]	137.9 (102)^b^	166.7 (110)**	165.2 (119)**	219.8 (113)^b^**	247.2 (179)**
EATV, [ml]	104.9 (53.4)	112.2 (62.8)**	114.7 (56.0)**	134.6 (65.3)**	146.6 (63.0)**
Increase in EATV [ml, vs. NGT]	N/A	7.3**	9.8**	29.7**	41.7**
EATA, [HU]	− 69 (8.0)	− 71 (6.0)**	− 69 (6.0)	− 71 (6.0)**	− 72 (7.5)**
Decrease in EATA [ml, group vs. NGT]	N/A	− 2**	0	− 2**	− 3**

Continuous data is presented as median and interquartile range (in brackets). Data with * or ** show statistically significant differences.

*p < 0.05 [Pearson Chi-square test for differences between glucose groups] **p < 0.05 [Independent Samples Kruskal–Wallis Test, group vs. NGT].

^a^13 + 4 + 8 + 1 + 2 = 28 cases of missing data in the groups.

^b^3 + 1 = 4 cases of missing data in the groups.

AUDIT alcohol use disorder identification test, HOMA IR homeostatic model assessment for insulin resistance, VAT visceral adipose tissue, EATV epicardial adipose tissue volume, EATA epicardial adipose tissue atteunation.

### Automatic quality checking of EAT segmentations

A total of 28 examinations were flagged by the automatic quality checking step in the image analysis, which were all manually reviewed and corrected. Of these, 13, or ~ 0.7% of the 1948 cases segmented, were visually deemed to be of unsatisfactory quality, requiring manual corrections to be included.

### Cohort characteristics

The cohorts showed mostly similar characteristics (Table 1), however with some notable exceptions; (i) there was a slight male predominance in the NGT and IGT groups and a slight female predominance in the other groups, (ii) BMI and waist were higher in the CGI and T2D group, (iii) the general level of education was slightly lower, while the prevalence of smoking was higher in the CGI and T2D groups, (iv) the use of lipid lowering medication was more widespread in the T2D group.

### EAT measurements

**Figure 1:**
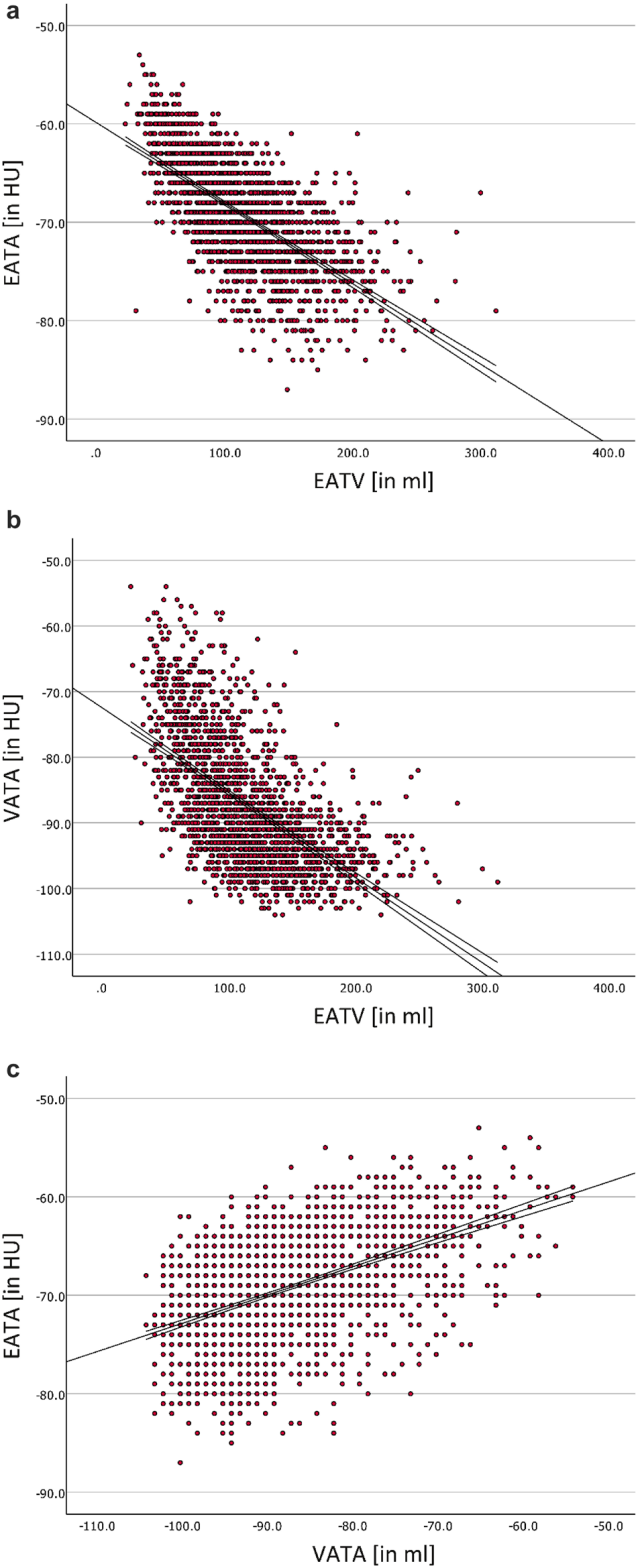
(**a**) Regression plot showing the relationship between epicardial adipose tissue attenuation (EATA) and epicardial adipose tissue volume (EATV). The regression line with its 95% CI is shown in black [B = 0.082, R = 0.665, p < 0.01]. (**b**) Regression plot showing the relationship between visceral adipose tissue attenuation (VATA) and epicardial adipose tissue volume (EATV). The regression line with its 95% CI is shown in black [B = (− 0.129), R = 0.589, p < 0.01]. (**c**) Regression plot showing the relationship between epicardial adipose tissue attenuation (EATA) and visceral adipose tissue attenuation (VATA). The regression line with its 95% CI is shown in black [B = (− 0.288), R = 0.514, p < 0.01].

The NGT group had a median EATV of 104.9 ml, the CGI group 134.6 ml (29.7 ml higher), and the T2D group 146.6 ml (41.7 ml higher), p < 0.01. Also, a significant positive correlation between EATV and the severity of glucose disorders in the order NGT-IFG/IGT-CGI-T2D was seen, Kendall´s tau correlation coefficient being 0.139, CI 0.106–0.172, p < 0.01. The CGI and T2D groups had a median EATA of -71 HU and -72 HU respectively, compared to − 69 HU in the NGT group, p < 0.01. A significant negative correlation between EATV and the severity of glucose disorders in the order NGT-IFG/IGT-CGI-T2D was seen, Kendall´s tau correlation coefficient being − 0.090; CI − 0.057, − 0.125; p < 0.01. EATA was also inversely correlated to EATV in the entire cohort (Fig. 1a), with lower attenuation values being more probable with increasing EATV (unstandardized B coefficient − 0.082; CI − 0.086, − 0.078; p < 0.01). VAT attenuation was also inversely correlated to EATV in the entire cohort, albeit with a steeper gradient (unstandardized B coefficient − 0.129; CI − 0.137, − 0.121; p < 0.01) (Fig. 1b). Consequently, EATA was positively correlated to VAT attenuation (unstandardized B coefficient 0.288; CI 0.266–0.309; p < 0.01), but the range of EATA is narrower than the range of VAT attenuation, such that an increase in VAT attenuation of nearly 10 HU corresponds to an increase in EATA of slightly less than 3 HU (Fig. 1c).

### Correlations between EATV, EATA and anthropometrics

As expected, the different anthropometrics showed a close correlation with EATV, ranging from R = 0.581 (between EATV and BMI) to R = 0.739 (between EATV and VAT area) in the NGT group. A somewhat weaker correlation was observed with EATA, ranging from R = 0.301 (between EATA and BMI) to R = 0.509 (between EATA and VAT area) in the NGT group. Similar values were found when all glucose groups were pooled together, with R = 0.572 to R = 0.743 and R = 0.257 to R = 0.468 between anthropometrics and EATV and EATA respectively.

### Selection of relevant confounders

In a sex stratified, stepwise logistic regression model including both anthropometrics and other confounders, VAT area showed the closest association with EATV and EATA in both men and women, while age showed the closest association with pre-diabetes (defined as IFG, IGT or CGI).

In the NGT group, age, sex, smoking and physical activity were independently associated with EATV when adjusting for VAT area. They were therefore included as confounders in the analysis of all glucose groups.

### Univariable versus multivariable analyses of the associations between EATV, EATA and glucose derangement

**Figure 2:**
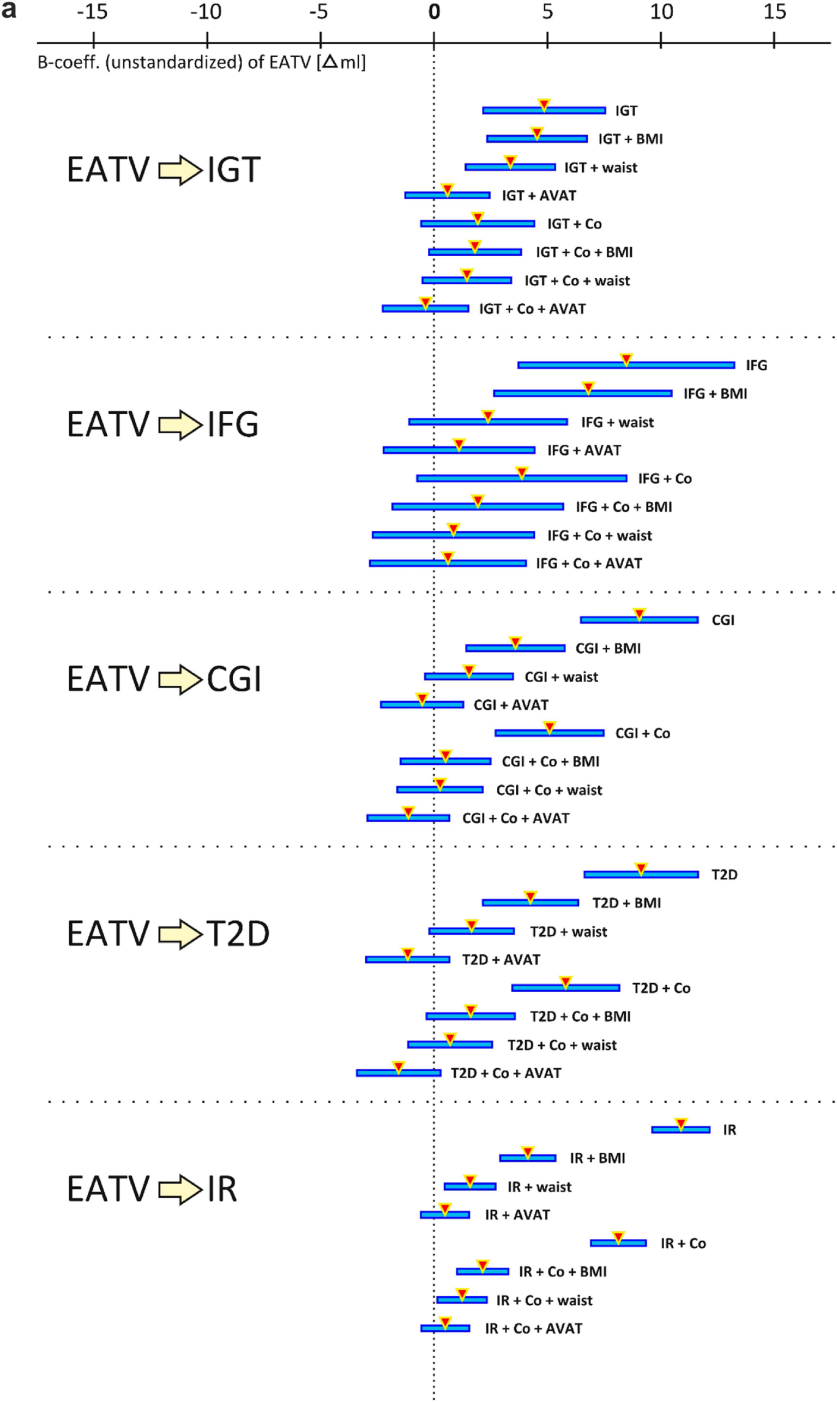

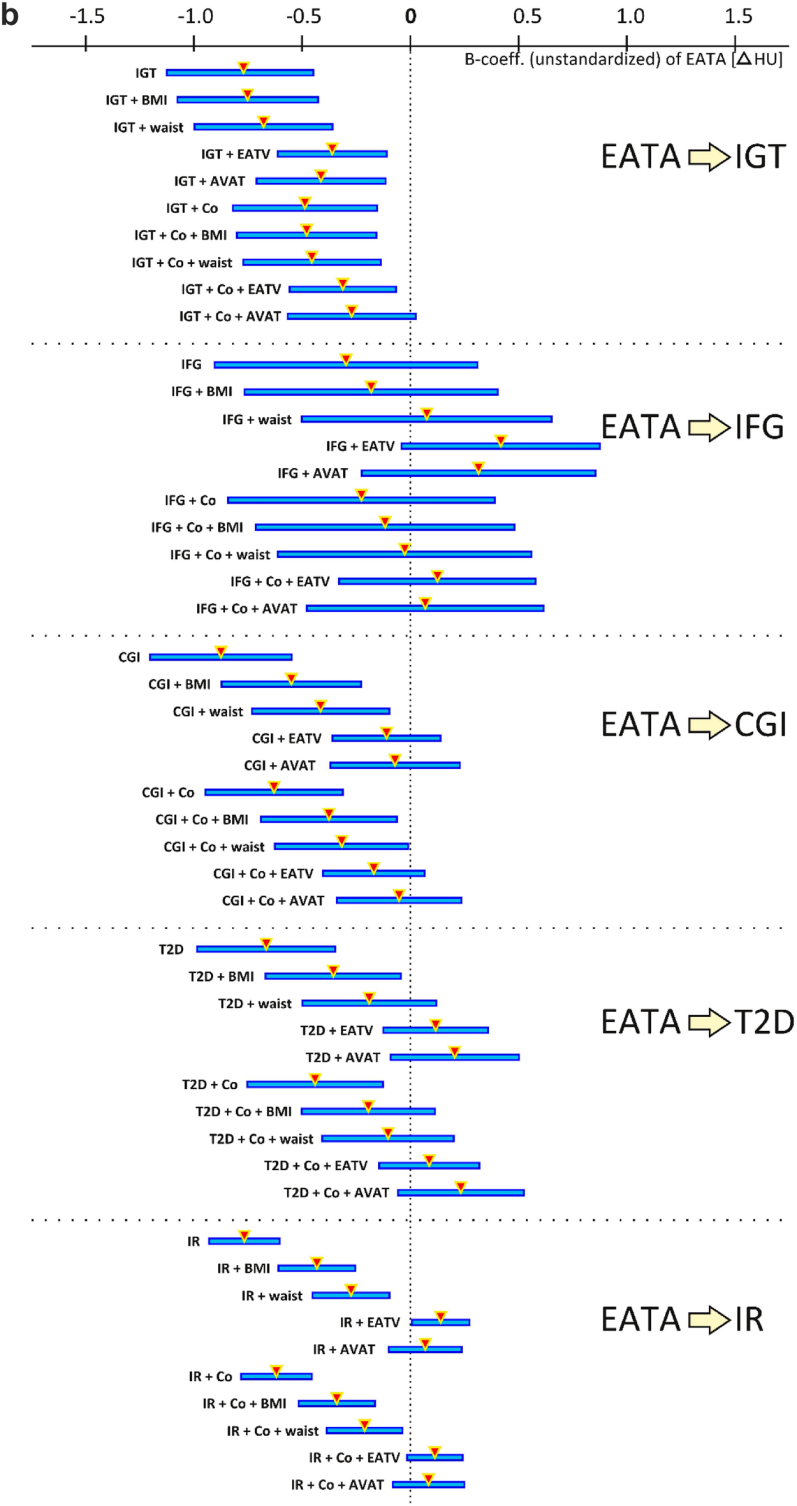
(**a**) Degree of association between epicardial adipose tissue volume (EATV) and the severity of derangement in glucose metabolism represented by the different stages of pre-diabetes (IGT, IFG, CGI), T2-diabetes (T2D) and insulin resistance (IR) when tested alone and in combination with co-variates (BMI, waist, abdominal VAT area) and the significant confounders age, sex, smoking and physical activity status (Co). The triangles and horizontal lines show the B-coefficient with its 95% confidence interval for the respective uni- or multivariable analyses, where touching or crossing the midline (zero) means loss of association for any of the given analyses. (**b**) Degree of association between epicardial adipose tissue attenuation (EATA) and the severity of derangement in glucose metabolism represented by the different stages of pre-diabetes (IGT, IFG, CGI), T2-diabetes (T2D) and insulin resistance (IR) when tested alone and in combination with co-variates (BMI, waist, abdominal VAT area) and the significant confounders age, sex, smoking and physical activity status (Co). The triangles and horizontal lines show the B-coefficient with its 95% confidence interval for the respective uni- or multivariable analyses, where touching or crossing the midline (zero) means loss of association for any of the given analyses.

In univariable analyses EATV showed a clear association with glucose groups, however, in multivariable analyses the association was lost after co-variates, other than BMI, and confounders were added to the analysis (Fig. 2a). EATA showed the strongest association in univariate analyses, and showed a slightly more independent association to the NGT, IGT and CGI groups in multivariable analyses. The association remained significant after addition of co-variates except for VAT area (Fig. 2b), when the independent association was lost in all groups other than IGT. No independent association was seen between EATA and the IFG group. Similarly to the association with glucose groups, both EATV and EATA showed an independent association with HOMA-IR until VAT area was added to the analysis as a co-variate (Fig. 2a,b).

### Association between abdominal VAT or SAT and IGT

To test whether the association between EATA and glucose groups was specific for EAT, we tested if similar associations were found for VAT and SAT. These tests were limited to the IGT group. Median attenuation of VAT and SAT in the NGT group was -88 HU (IQR: 13) and -106 HU (IQR: 5) respectively while attenuation of VAT and SAT in the IGT group was -92 HU (IQR: 12) and -107 HU (IQR: 4) respectively. In multivariable analyses IGT was associated with VAT attenuation (p < 0.05) but not with SAT attenuation (p = 0.394) when adjusting for the area of the fat depot as well as co-variates.

### Overview of relevant literature

Important studies in the literature, comprising cohorts of sufficient numbers, or employing methods of interest are presented for reference purposes (Table 2). Six studies, comprising a total of 3508 participants, were found, where EATV in T2D and/or pre-diabetes was compared to controls (totally 2423) with normal glucose homeostasis. Among these, three studies, comprising totally 1640 participants, included pre-diabetic individuals (totally 356 cases with pre-diabetes), however, without making any distinction between the various stages of pre-diabetes. Three studies, comprising totally 1868 participants, compared non-diabetic and diabetic participants (totally 479 cases with T2D). The results, although showing large spread in the measured EATV, consistently show an increase in EATV in T2D and pre-diabetic states. Interestingly, no studies presenting EATA data in pre-diabetes were found. One study by Lin et al. focused on the metabolic syndrome, but deserves mention, since it employed an automatic technique for EATV and EATA analysis similar to ours, and also represents the largest cohort, where EATA was measured. Two studies deserve mention, measuring other aspects of EAT amount than EATV; one study by Rado et al., which included pre-diabetic individuals, found an increased EAT area on MRI images in both pre-diabetes and T2D, and a study by Chun et al., which showed increased EAT thickness in T2D.

**Table 2:** Overview of the most important studies in the field with regards to cohort size and/or techniques applied, arranged in inverse chronological order.

Authors	Year	Study population (total n)	Groups	n	EATV (ml)	EATA (HU)	EATT (mm)	EATAr (cm^2^)	Method
Lin et al.^34^	2021	EISNER (2068)	MetS +	280	114.1 [90.7–147.8]	− 76.9 ± 4.6			NCCT, automatic
			MetS−	1788	73.7 [53.7–98.7]	− 73.4 ± 4.6			
Wang et al.^12^	2019	Shanghai, cohort study (668)	NFG	468	117.34 [82.67–167.19]				NCCT, manual
			IFG/IGT	83	173.21 [139.67–219.53]				
			T2D	117	190.64 [138.33–244.82]				
Rado et al.^44^	2019	KORA, MONICA substudy (372)	NGT	220				7.7	MRI, semi-automatic, from 4-chamber-view
			Pre-T2D	100				9.2	
			T2D +	52				10.3	
Milanese et al.^17^	2018	Parma, retrospective (1379)	T2D +	338	112.87* [IQR 68.07]	− 80.78 ± 6.06			CCT, semi-automatic
			T2D−	1041	82.62* [IQR 62.17]	− 78.19 ± 5.27			
Chun et al.^16^	2015	Korea, cross-sectional (1048)	T2D +	141	N/A	N/A	17.6 ± 6.7		NCCT, manual, around LMCA
			T2D−	907	N/A	N/A	14.4 ± 5.9		
Groves et al.^20^	2014	California (362)	T2D +	92	118.6 [75.6–161.6]				CCT, manual
			T2D-	270	70.0 [26–114]				
Yang et al.^11^	2013	Taiwan (562)	NGT	357	68.2 [42.7–93.7]				NCCT, manual
			Pre-T2D	155	86.8 [59.9–113.7]				
			T2D +	50	91 [66.9–115.1]				
Versteylen et al.^5^	2012	Utrecht (410)	T2D +	83	98 [57–139]				CCT, manual
			IFG	118	92 [53–131]				
			NFG	209	75 [41–109]				
Wang et al.^18^	2009	Taiwan, case–control study (127)	T2D +	49	166.1 [105.5–226.7]				NCCT, manual
			T2D−	78	123.4 [81.6–165.2]				

*T2D* type-2 diabetes, *IFG* impaired fasting glucose, *IGT* impaired glucose tolerance, *pre-T2D* pre-diabetes, *NFT* normal fasting glucose, *NGT* normal glucose tolerance, *MetS* metabolic syndrome, *EATV* epicardial adipose tissue volume, *EATA* epicardial adipose tissue attenuation, *EATT* epicardial adipose tissue thickness, *EATAr* epicardial adipose tissue area, *VAT* visceral adipose tissue, *NCCT* non-contrast computed tomography, *CCT* contrast computed tomography, *MRI* magnetic resonance imaging.

## Discussion

In this report, we present CT derived data on EAT volume and attenuation from a cohort of 1948 participants, representing various states of glucose metabolism ranging from normal over pre-diabetes to fully developed type-2 diabetes.

In univariable analyses there was a gradual increase in EATV and a concomitant decrease in EATA with the severity of glucose disorders. However, both EATV and EATA exhibited strong co-variation with BMI, waist-circumference and abdominal VAT area. Multivariable linear regression analyses were used to investigate whether EATV or EATA were associated with glucose disorders independently of other anthropometrics. It was evident from these analyses that it is difficult to discern a clear independent role of EATV and EATA in disease development, especially when adjusting for the size and characteristics of the abdominal VAT depot. EATA showed a tighter and somewhat more independent association with early pre-diabetes/impaired glucose tolerance (IGT) than EATV. Further, since pre-diabetes is associated with impaired insulin secretion and/or insulin resistance (WHO Report, 2006) we also included HOMA-IR in the analysis and found that an increase in IR was also associated with increased EATV and decreased EATA.

### EATV gradually increases with the severity of changes in glucose metabolism

We found a gradual increase in EATV in the different groups representing the various stages of disease progression to T2D. Numerous studies, predominantly employing either echocardiography^13–15^ or computed tomography (CT)^5,16–18^ have presented convincing evidence of EAT expansion in T2D relative to controls: the largest cohorts of individuals, where EAT thickness was measured comprise 1004 (770 with T2D) individuals examined by echocardiography^15^, and 1048 (141 with T2D) individuals examined by CT^16^, while the largest cohort where EATV was measured in CT comprise 1379 (338 with T2D) individuals^17^. EATV was found to be increased^5,11,12^ to a lesser extent in pre-diabetic individuals, than individuals with T2D in three studies. Although all of these represent selected populations, do not separate IFG from IGT, and are relatively small, comprising 410 (118 with IFG), 562 (155 with pre-diabetes) and 668 (83 with IFG/IGT) individuals respectively, they suggest that the changes in EATV from normoglycaemia to T2D might reflect a gradual process. Our present study is the first to subdivide pre-diabetic individuals into IFG, IGT and CGI groups reflecting the various stages of disease development, and also the largest to date, comprising 863 individuals with pre-diabetes. It seems, from our data, that EATV expansion is continuous over the categories. Some caution is however warranted in drawing far reaching conclusions on the independent role of EATV in disease development, as other visceral fat depots also have a tendency to expand in parallel in the process leading to established T2D^12,39^. It is clear that both BMI and waist circumference, which are easily measured, give an indication of the degree of EATV expansion, as does the VAT area, which is its closest approximation^23,39^. Up to 40% of the variation in EATV could be explained by various anthropometrics in 1400 individuals representing a cross-section of the population in our recent work^24^. In the present study, which is the first on EAT in pre-diabetes and T2D fully adjusting for anthropometrics, EATV was independently associated with pre-diabetes only in relation to BMI, a general measure of adiposity. There was no independent association between EATV and pre-diabetes when adjustments were made for waist circumference or VAT area, underscoring that abdominal visceral fat volume seems indeed to expand in parallel with EATV in individuals developing disorders of glucose metabolism.

### EATA gradually decreases with the severity of changes in glucose metabolism

We found a gradual decrease in EATA in the different groups representing the various stages of disease progression to T2D. It is an interesting perspective, that the radiodensity, or attenuation of EAT might provide more selective information on the metabolic state in an individual than EATV. Embryologically and physiologically, EAT shares properties with other visceral fat depots, albeit with some important differences: there is no fascia separating it from the myocardium, the coronary arteries are directly exposed to metabolites and secretions produced in the EAT, the adipocytes in EAT are generally smaller showing increased lipid turnover^7^, and finally, EAT shows a varying degree of beige-fat like features^40^. There are only a few studies that have measured EATA in T2D (Table 2). Both Franssens^25^ and Milanese^17^ found that EATA was decreased in patients with established T2D, consistent with our data. We could expand on their findings and demonstrate a gradual reduction in EATA over the different stages of pre-diabetes and insulin resistance. The reduced EATA in pre-diabetes was independent of concomitant changes in BMI and waist circumference, but not VAT area. This differs from the findings in EATV, and may suggest that EATA has a more independent role in diabetes related disease development than EATV.

An increased density of triglycerides in the epicardial fat depot in response to chronic dietary overload^29^ could be an explanation to the reduced attenuation of EAT seen in pre-diabetes and T2D. However, the situation is probably more complex. There is increasing evidence, that EATA is modulated by several factors, not only the regulation of brown adipose tissue-like features, but also local inflammation around the coronary arteries, i.e. in the pericoronary adipose tissue (PCAT)^41–43^. Augmented cellularity due to inflammation or an increase in extracellular matrix seen in fibrotic (post)inflammatory conditions^29^ can increase EATA. There is also a gender- and age-specific correlation, as shown by Franssens et al., EATA being lower in women, and lower with advancing age^25^. Consequently, the exact mechanism leading to lower EATA with gradual metabolic derangement can only be speculated on.

### Reduction of attenuation is also seen in abdominal VAT but not in SAT

To complement our analyses on EATA, we analyzed tissue attenuation in abdominal VAT and subcutaneous adipose tissue (SAT). Interestingly, the exact same pattern of reduced attenuation in pre-diabetes and T2D was seen in VAT but not in SAT. This suggests that EATA and VAT, both visceral fat depots, share important characteristics, but behave distinctly differently from SAT.

### Confounders

A number of confounders need to be addressed. In a prospective study, Nerlekar et al. found that advancing age seemed to decrease EATA independently^30^ of other factors such as the occurrence of coronary atherosclerosis or diabetes^30^. Franssens et al. also found EATA to be lower with advancing age^25^, but also lower in women, the former possibly stemming from a natural reduction of brown adipose tissue^31^ in ageing. Further, cholesterol metabolism could influence EATA: Raggi et al. found in a recent work that one year of cholesterol-lowering therapy reduced EATA, an effect not seen in subcutaneous adipose tissue^32^. It is known that an increase in metabolically active brown adipose tissue can increase EATA^26,27^. Archer et al. found seasonally dependent^28^ differences in EATA in retrospective analyses of 597 cardiac CT examinations. In our analyses, on the contrary, neither treatment for hyperlipidemia nor season (quarter of year) of CT examination were found to affect EATA. Sex, age, physical activity and smoking were however found to affect EAT results significantly, and were therefore included in the multivariable analyses as confounders.

### Limitations and strengths

This study focused on recruiting a large group of participants representing different stages of glucose derangements from normal via impaired fasting glucose and impaired glucose intolerance to fully developed T2D. Nevertheless, the group sizes are relatively modest, especially the T2D-group, resulting in broad confidence intervals and lack of power. It is important to note that the T2D cases were all newly diagnosed, i.e. in the early phase of disease development and cannot be easily compared to subjects with long-standing and treated T2D as used in previous studies by Franssens^25^ and Milanese^17^ et al. addressing EATV and EATA. The T2D cases were therefore naïve to any specific anti-diabetic treatment, limiting confounding effects. Since our data is cross-sectional, no inference can be made on the causal role of EATV and EATA in disease development. Nevertheless, our present study is still one of the largest with regards to EAT data on pre-diabetic individuals, obviously an area which has been scarcely covered.

Another potential limitation would be that our EAT data was automatically generated, with manual quality check only of segmentations flagged as potentially flawed by the fully automated model. However, our automatic quality check showed very good performance in our evaluation, comprising totally 1800 CT-examinations (1400 cases used in the validation process of segmentation quality^24^ and 400 used to test the performance in the actual cohort) acquired with identical scanners and technical protocols. We have no reason to believe, that the performance would be inferior to the performance previously seen in images with identical technical parameters, especially since the manual quality check of the flagged segmentations showed small errors in the majority of the cases, which we interpret as proof of the feasibility of the filter used. Also, an obvious strength with a fully automated analysis of the CT images, apart from the time-efficiency is the reproducibility, which should be superior to manual analysis in all instances.

### Clinical potential

In light of our findings, it seems that EATV and EATA is indeed altered in pre-diabetes and diabetes, however, not independently from most other fat depots and especially not from the abdominal visceral fat depot. There is convincing evidence in the literature that visceral fat is of pathophysiological importance in metabolic and cardiovascular disease development and it is therefore interesting that data from the epicardial fat depot appears to change in parallel with visceral abdominal fat. We have shown that low-dose, non-contrast cardiac CT images can be used to automatically and reliably measure EATV and EATA, and with small adaptations this model can be used on any standard non-contrast cardiac CT examination designed to assess coronary artery calcifications. In a cardiac prevention program, this easily accessible reflection of visceral fat quantity and characteristics could give important information on the risk of both developing metabolic disease and its cardiac complications.

## Conclusion

The current work has presented a large cohort of subjects with disorders of glucose metabolism ranging from normal glucose tolerance to T2D, where automatic measurements of EATV and EATA have been performed. We found that EATV is increased and EATA reduced in pre-diabetes, T2D and IR, however, the significant co-variation with other anthropometrics, especially abdominal VAT, makes it difficult to discern an independent role of this fat depot in disease development. The current results do not exclude a pathophysiological role of epicardial fat, but future studies need to adjust for anthropometrics and confounders, and most likely also analyze patterns of adipose volume and attenuation locally around or in the studied target organ.

## Data Availability

The datasets used and/or analyzed during the current study available from the corresponding author on reasonable request.
